# Overcoming Chemotherapy Resistance in Cutaneous T-Cell Lymphoma: A Successful Case of High-Dose Radiotherapy Management

**DOI:** 10.7759/cureus.43959

**Published:** 2023-08-23

**Authors:** Fadila Kouhen, Kenza Oqbani, Hanae El Gouach, Meriem Ahnach, Zineb Dahbi

**Affiliations:** 1 Laboratory of Neurosciences and Oncogenetics, Department of Radiotherapy, Mohammed VI Center for Research and Innovation, International University Hospital Sheikh Khalifa, Mohammed VI University of Sciences and Health (UM6SS), Casablanca, MAR; 2 Department of Pathology, International University Hospital Sheikh Khalifa, Mohammed VI University of Sciences and Health (UM6SS), Casablanca, MAR; 3 Department of Hematology, International University Hospital Sheikh Khalifa, Mohammed VI University of Sciences and Health (UM6SS), Casablanca, MAR; 4 Department of Radiotherapy, International University Hospital Sheikh Khalifa, Mohammed VI University of Sciences and Health (UM6SS), Casablanca, MAR

**Keywords:** systemic treatment, excellent outcome, cutaneous t-cell lymphoma, radiotherapy, chemotherapy resistance

## Abstract

The management of refractory cutaneous T-cell lymphoma (CTCL) is challenging and requires a multimodal approach. Radiotherapy is one of the treatment options used in managing CTCL, particularly for localized disease or as a palliative measure to control symptoms in advanced cases.

The rarity of the disease makes it difficult to conduct extensive clinical trials and gather sufficient data on the most effective treatment approaches. Lymphocytes are among the most sensitive cells to radiation's damaging effects. Because of this sensitivity, radiation therapy can be an effective treatment.

This case illustrates the efficacy of radiotherapy and its potential as an effective treatment alternative for a severe and resistant CTCL to systemic therapy in a 61-year-old Moroccan patient. The patient underwent curative high-dose radiation therapy, utilizing three-dimensional conformal radiation therapy. At the 19-month follow-up post-radiotherapy, no evidence of local recurrence, either clinically or radiologically, was observed, and the patient maintained a good quality of life with unrestricted mobility of his arm.

## Introduction

Cutaneous T-cell lymphoma (CTCL) is a rare group of non-Hodgkin lymphomas characterized by the clonal proliferation of skin-homing malignant T lymphocytes and, in some cases, natural killer cells [1]. The disease primarily affects the skin, and its presentation can vary widely, ranging from limited skin involvement to more widespread skin lesions.

The exact cause is not well understood, but it is thought to involve a combination of genetic factors and environmental triggers [2]. The diagnosis is based on a combination of clinical features, skin biopsies, and immunohistochemical and molecular studies to identify the presence of abnormal T-cell or NK cell clones. There are different subtypes of CTCL, with mycosis fungoides (MF) and Sézary syndrome being the most common ones.

Management of CTCL typically involves a multimodal approach tailored to the specific subtype and stage of the disease. The different therapeutic approaches are aimed at controlling the disease, alleviating symptoms, and improving the patient's quality of life. Lymphocytes, being highly active and rapidly dividing cells, are particularly sensitive to radiation, making it an effective treatment for conditions involving these cells, such as MF [3]. Additionally, radiation therapy is advantageous compared to other skin-directed therapies because it can penetrate and treat thicker plaques and tumors effectively. This characteristic makes it suitable for cases where the lesions are more extensive or deeply rooted in the skin.

The decision to use radiotherapy as a treatment option for CTCL depends on various factors, including the stage of the disease, the number and size of skin lesions, the patient's overall health, and their response to previous treatments. Often, radiotherapy is used in combination with other therapies, such as topical treatments, systemic therapies, or phototherapy [4].

Our case demonstrates the potential of radiotherapy as an effective treatment option for aggressive and refractory cutaneous lymphomas. This case also underscores the importance of personalized treatment approaches and the need to consider alternative options when conventional therapies are ineffective.

## Case presentation

**Figure 1:**
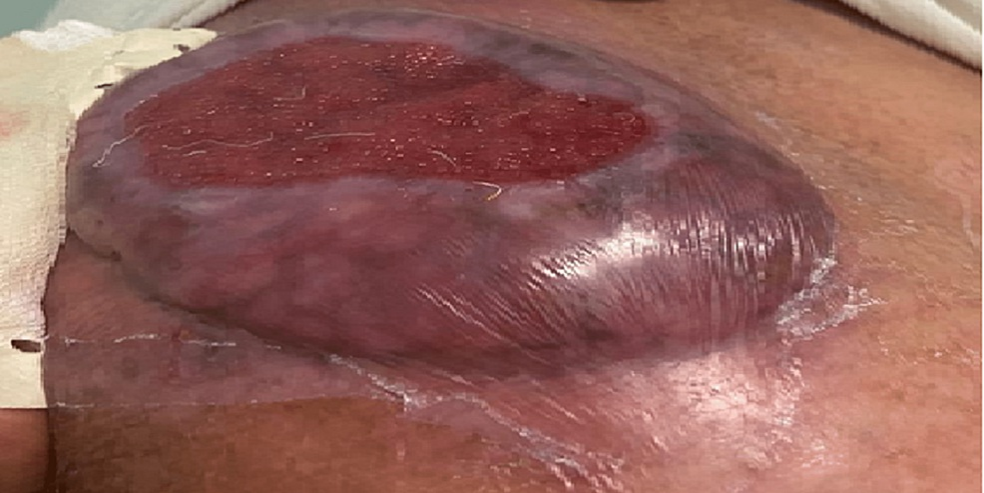
The budding lesion on the right shoulder before radiotherapy.

A 61-year-old Moroccan male with no significant past medical history presented to our department with a painful, solitary raised growth on the right shoulder. The growth had developed over the past six months and had been steadily increasing in size (Figure 1).

On physical examination, the general state was normal. The lesion was painless. It measured approximately 10 cm in diameter and was oval shaped with irregular borders. There was no redness, inflammation, or swelling in the surrounding skin. No lymph nodes were palpable, and no other suspicious skin lesions were observed. The lesion did not affect his daily activities.

**Figure 2:**
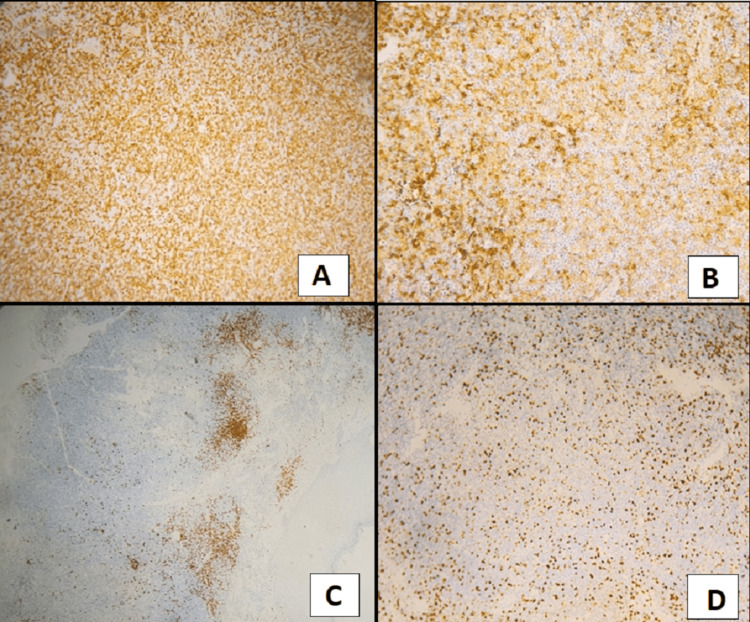
Mycosis fungoides immunohistochemistry. (A) CD3 was widely positive. (B) Neoplastic cells expressed variably CD4 and weakly CD8. (C) Negative staining for anti-CD20. (D) Ki-67 stain showed a low-to-medium proliferation rate (25%).

A biopsy was performed, and the microscopic examination revealed a cutaneous tissue with diffuse dermal infiltrates, without epidermotropism. The tumor was composed of a variable proportion of small- and medium-sized cells. There were also some rare large cells with pleomorphic nuclei. Tumor cells were positive for CD3 and CD4 and negative for CD8 and CD30 antibodies. The immunohistochemical stains had shown a diffuse and strong positivity for CD3 and a heterogeneous positivity for CD4 and CD8. All other markers studied were negative, including CD20, CD15, and CD138 antibodies. The Ki-67 proliferation index was estimated at 25%. In light of microscopic and phenotypic results, the diagnosis of an advanced MF was confirmed (Figure 2).

A fluorodeoxyglucose (FDG) positron emission tomography conducted on this patient revealed a hypermetabolic skin lesion at clinically affected sites, with no indications of systemic disease. The bone marrow biopsy was negative for lymphomatous involvement.

The case was discussed in a multidisciplinary tumor board, and the decision to initiate chemotherapy was agreed upon.

The patient received two cycles of the cyclophosphamide, doxorubicin, vincristine, and prednisone (CHOP) regimen without clinical improvement. Following the latest update of the European Organization for Research and Treatment of Cancer consensus recommendations for treating MF/Sézary syndrome, and considering the absence of accessible targeted immunotherapy, a second-line chemotherapy regimen involving dexamethasone, Ara-cytarabine, cisplatin (DHAP) was initiated. His chemotherapy course was complicated by acute renal failure with febrile neutropenia, sepsis, and clinical deterioration, requiring hospitalization and discontinuance of treatment.

**Figure 3:**
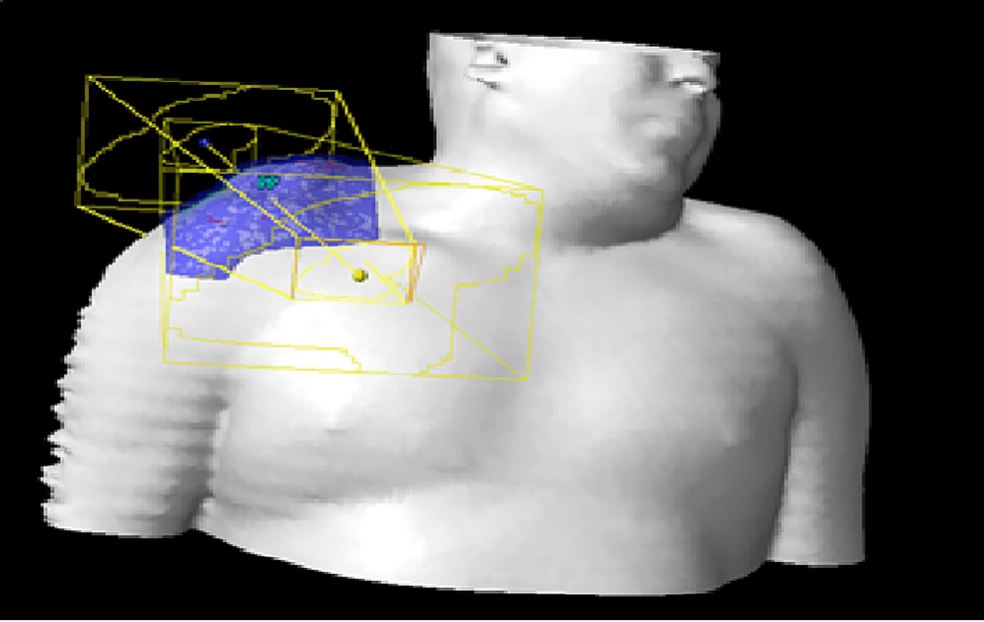
Graphical representation of 3D irradiation fields treatment with a 5-mm-thick tissue equivalent bolus (the patient consented to include his photos and clinical case as a case report). 3D, three-dimensional

**Figure 4:**
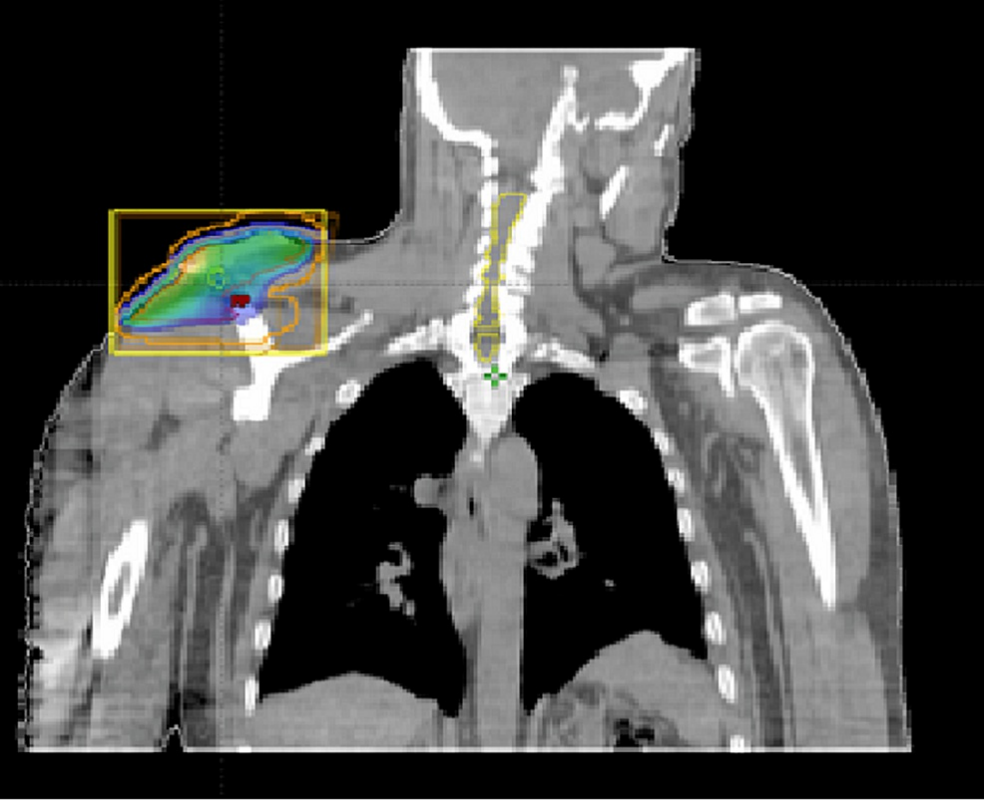
Dosimetric planning with opposite radiotherapy fields: a coronal scan image.

Owing to the insufficient response and significant toxicity of systemic therapy, the proposition of 3-Dimensional Conformal Radiotherapy as a salvage treatment arose. This approach utilized a 6 MV photon beam administered via the Truebeam linear accelerator (Varian, Palo Alto, CA, USA), with a total prescribed dose of 40 Gy. The patient was treated five days per week with daily dose per fraction of 2 Gy. Two lateral fields were employed with a 5-mm-thick daily tissue equivalent bolus (Figures 3-4) while protecting the ipsilateral humeral head. The right arm was immobilized with a Vac-lok immobilization device.

**Figure 5:**
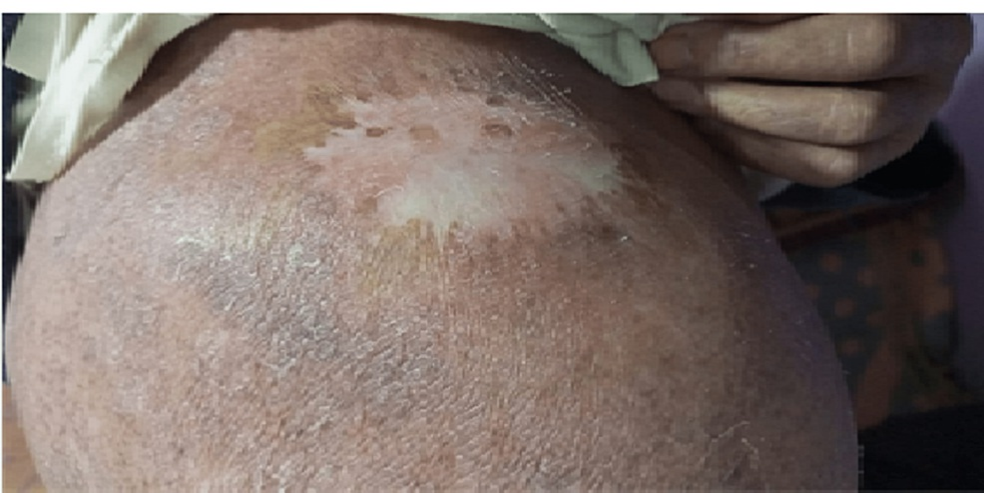
Complete clinical response three months after radiotherapy.

After a 19-month follow-up period, there was a complete response, and the patient shows no clinical evidence of recurrence (Figure 5). An 18F-FDG PET scan was performed at 6, 12, and 18 months after treatment, and no evidence of recurrence, whether local or distant, was detected. 

The patient's skin has fully healed to a state of normal softness and smoothness after one month following the completion of radiotherapy. There were no notable mobility issues or limitations due to the treatment.

## Discussion

CTCL is a group of extra-nodal non-Hodgkin lymphoma defined by clonal proliferation of skin-homing malignant T lymphocytes and natural killer cells [5]. It represents 4% of all non-Hodgkin lymphomas and 75% to 80% of all primary cutaneous lymphomas. The median age at the diagnosis varies between 55 and 60 years [6]. The 2017 World Health Organization (WHO) classification divided CTCL into various subtypes, and this classification was further updated in 2018 by the WHO-European Organization for Research and Treatment of Cancer (WHO-EORTC). MF is the most common subtype, accounting for approximately 60% of all CTCL cases [7].

Symptoms of CTCL can vary depending on the stage and subtype of the disease but often include skin rashes, red patches, plaques, and tumors. Some patients may also experience itching, scaling, and skin ulceration [8].

Many factors are involved in determining effective treatment strategies for each patient, including clinical stage at presentation, type of skin lesion, as well as response to therapy. New therapies are constantly tested for more effective treatments and fewer side effects [9].

The stages of MF are classified based on the extent and severity of skin involvement, and the treatment approach may vary accordingly.

Stage I MF is characterized by limited skin involvement with patches and plaques. In these cases, topical therapies are often the first line of treatment such as topical corticosteroids, topical nitrogen mustard, and topical retinoids. In more advanced stages of the disease, such as stage IIB or greater, or when the condition fails to respond to topical treatments, systemic therapies are generally recommended [10,11].

Due to the extreme radiosensitivity of lymphocytes even with low doses, radiation therapy plays a central role in the treatment of CTCL, either as the exclusive treatment or as part of a multimodal approach [3]. Solitary or localized lesions can be successfully treated with local radiation of 40 Gy, although palliative radiation is effective in treating large, refractory, and symptomatic diseases.

Traditionally, multiple-fraction radiation therapy has been used in CTCL treatment, where patients receive smaller doses of radiation over several treatment sessions. However, some studies have explored the use of single-dose radiation as an alternative approach. Single-dose radiation has advantages such as reducing the overall treatment burden for patients and potentially providing comparable palliative effects [12]. Thomas et al. conducted a retrospective review indicating that the use of a single dose of radiation (700-800 cGy) as a treatment for CTCL resulted in excellent palliation in patients, with 94.4% of the lesions achieved a complete response, while an additional 3.7% showed a partial response, indicating a significant overall response rate [13]. This suggests that a single, higher dose of radiation may be as effective as multiple-fraction radiation treatments for palliative care in this particular group of patients.

Special techniques such as total skin electron beam therapy (TSEBT) using a six-field technique might be necessary to cover more extensive lesions [14].

TSEBT is considered an effective modality for treating CTCL because it can deliver high doses of radiation to the entire skin surface, penetrating the outer layers of the skin to reach the affected T-cells in the deeper layers [15]. By targeting the entire skin, TSEBT aims to treat both visible and invisible skin lesions.

The goal of TSEBT is to reduce or eliminate skin lesions and achieve a partial or complete response in CTCL patients. The treatment is usually delivered in multiple sessions over several weeks. TSEBT is often reserved for cases where other treatments have not been successful or when there is widespread involvement of the skin [16].

In the past, TSEBT was typically delivered in fractions of 1.5 to 2 Gy per session, with total doses of ≥30 Gy. This high-dose regimen was effective in treating CTCL but was also associated with significant side effects and morbidity [17].

However, since 2011, there has been a shift toward using low-dose TSEBT (<30 Gy) for CTCL treatment. Several studies have been published supporting the use of lower doses due to similarities in overall response rates when compared to higher dose regimens (≥30 Gy) [18]. Additionally, low-dose TSEBT is associated with lesser toxicity and shorter treatment times.

Side effects of radiation therapy vary largely, depending on the dose, number of fractions, and the radiation's technique. It can include erythema, desquamation, atrophy, and skin dryness.

## Conclusions

This case report supports the use of radiotherapy as an effective treatment for CTCL in certain patients, particularly those with advanced-stage or refractory disease.

With advancements in radiotherapy techniques and combination therapies, the goal of achieving a cure while managing treatment-related toxicities becomes more attainable. However, treatment decisions should always be individualized and made in consultation with a specialized medical team to provide the best possible outcomes for each patient.

It is important to emphasize the need for more in-depth investigation and evaluation to further refine and optimize treatment protocols for this complex disease.
